# Hospital Practices for Respiratory Isolation for Patients With Suspected Tuberculosis and Potential Application of Prediction Models

**DOI:** 10.7759/cureus.32294

**Published:** 2022-12-07

**Authors:** Joshua Davidson, Bryan Chesen, Samir Kumar, Daniel J Shayowitz

**Affiliations:** 1 Pulmonary and Critical Care Medicine, BronxCare Health System, New York, USA; 2 Pulmonary and Critical Care Medicine, State University of New York Downstate Health Sciences University, Brooklyn, USA; 3 Pulmonary and Critical Care Medicine, Mount Sinai Health System, New York, USA; 4 Internal Medicine, State University of New York Downstate Health Sciences University, Brooklyn, USA

**Keywords:** : tuberculosis, airborne infection control, hospital infection control, airborne isolation, prediction tools, decision tree, flexible fiberoptic bronchoscopy (ffb), active pulmonary tuberculosis, hospital isolation

## Abstract

Hospitalized persons with suspected pulmonary tuberculosis (PTB) are placed in airborne isolation to prevent nosocomial infection, as recommended by the Centers for Disease Control and Prevention (CDC). There is significant evidence that clinicians overuse this resource due to an abundance of caution when confronted with a patient with possible PTB. Many researchers have developed predictive tools based on clinical and radiographic data to assist clinicians in deciding which patients to place in respiratory isolation. We assessed the isolation practices for an urban hospital serving a large immigrant population and then retrospectively applied seven previously derived prediction models of isolation of PTB to our population.

Our current clinical practice results in 76% of patients with PTB being placed in isolation on admission. However, 208 patients without PTB were placed in isolation unnecessarily for a total of 584 days. Four models had sensitivities greater than 90%, and two models had sensitivities of 100%. The use of these models would have potentially saved more than 150 days of patient isolation per year.

## Introduction

Tuberculosis (TB), caused by the bacterium Mycobacterium tuberculosis, is one of the most deadly pathogens in human history [1]. The most common (~85%) site of infection is the lungs (pulmonary), though the disease is known to affect almost all tissues in the human body (extrapulmonary). Mycobacterium tuberculosis is highly contagious and spread through the air in respiratory droplets. Thus, the standard of care for patients who are hospitalized with suspicion of pulmonary tuberculosis (PTB) is to place them in respiratory isolation to prevent nosocomial infection [2]. Due to the concern of missing a potential case of TB, physicians often employ a low threshold of suspicion to isolate patients.

Respiratory isolation requires a resource-intense utilization of healthcare, for which the Centers for Disease Control and Prevention (CDC) has provided extensive guidelines [2]. The structural aspects include a negatively pressurized room with an anteroom to create a double barrier between the desired isolation area and the rest of the hospital [3]. The isolation room should have an independent exhaust system with high-efficiency air filters and ultraviolent germicidal irradiation of the air. The hospital must supply adequate personal protective equipment (PPE), including N95 masks. Personnel is another major area of expense as healthcare workers must all be trained in proper management of isolation of patients. The CDC also recommends cough etiquette training. Hospitals should have a TB control team, which may be part of an infection control department. Finally, sputum sample collection and testing are necessary; testing may be performed on-site or at a central mycobacterium laboratory [2].

Respiratory isolation of patients leads to increased hospital expenses and increased length of stay [4]. It may also negatively affect patients, creating stress and decreased morale due to lack of human contact [5]. The COVID-19 pandemic has led to many patients being placed in respiratory isolation, placing further demands on a limited resource.

The CDC recommends that patients suspected of having PTB be placed in respiratory isolation and sputum samples collected for acid-fast bacilli (AFB) staining and culture. AFB staining involves staining the sputum sample and examining it under a microscope for the presence of acid-fast organisms. The CDC recommends that three samples be collected at least eight hours apart, including at least one morning sample to diagnose PTB [2]. Morning samples may offer the highest yield as respiratory secretions can pool in the large airways while patients are supine. Patients may be taken off isolation when three consecutive AFB stains are negative for the presence of TB or when TB is no longer clinically suspected. However, Mycobacterium tuberculosis may take up to six weeks to grow on culture media. Only patients with high mycobacterial loads will have a positive sputum AFB stain. Between 30% and 50% of patients with PTB will have smear-negative sputum [6]. The subset of patients who have smear-positive sputum are thought to be the most highly contagious; therefore, when a patient with PTB has three consecutive negative sputum AFB smears, the standard of care is to deem them safe for discontinuation of respiratory isolation precautions.

Some patients are unable to expectorate enough sputum for sampling. These patients may require more invasive procedures, often bronchoscopy, to make the diagnosis of PTB. However, the role of routine bronchoscopy in patients with smear-negative sputum and suspicion of PTB is unclear.

Predicting which patients have PTB based on clinical and radiographic findings has been an area of significant clinical inquiry. This has led to the development of many prediction algorithms for deciding which patients with suspected PTB to be placed in respiratory isolation, to minimize the burden both on patients and the healthcare system [7]. We sought to retrospectively evaluate the impact of several previously published clinical-radiographic predictive algorithms on a New York City hospital servicing a large immigrant and underserved population.

## Materials and methods

**Figure 1:**
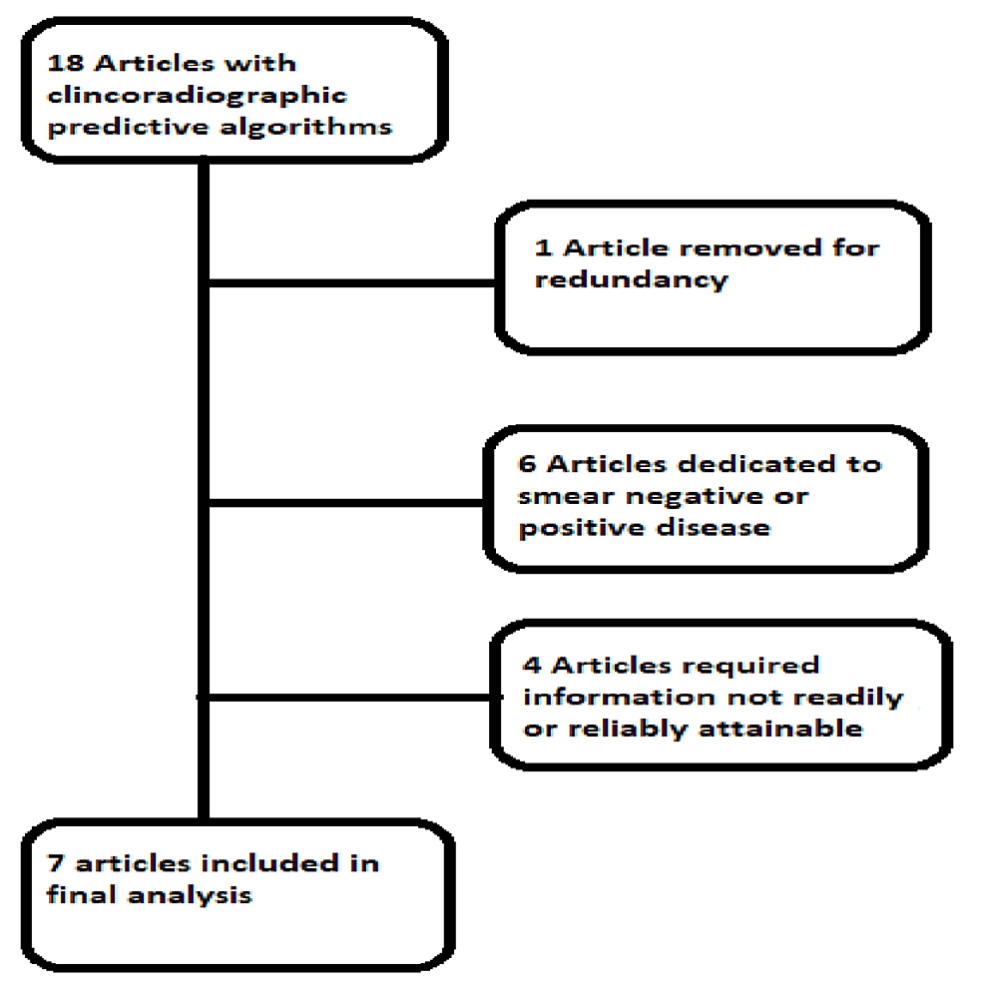
Articles selection. Figure credits: Joshua Davidson

We searched MEDLINE for papers with prediction algorithms using the search MeSH terms “Tuberculosis/diagnosis,” “Tuberculosis/diagnostic imaging,” and “Mycobacterium tuberculosis/diagnosis,” or “Mycobacterium tuberculosis/isolation and purification” in combination with the terms “Decision Trees,” “Predictive Value of Tests,” “clinical predictors,” or “logistic model.” We then reviewed the citations of the selected articles for additional suitable articles. We identified 18 articles with prediction algorithms (Figure 1). One article was removed as it served to confirm/verify the results of a previously published derivation work [6]. Six of the articles focused on a subset of patients based on smear data, while our analysis was based on culture data. Five of these articles focused on smear-negative TB patients, and one article focused on smear-positive patients [8-13]. Finally, four of the articles relied on subjective information or data such as CT scans, which we felt would not be necessarily available in many of our patients [14-17]. Seven articles were available for analysis and comparison for a retrospective data set [18-24].

We reviewed patients with confirmed PTB aged 12 years or older, admitted to an urban safety net hospital (King’s County Hospital Center, Brooklyn, NY, USA) servicing a large immigrant and underserved population between 2014 and 2018 and compared them to patients who were placed in respiratory isolation for suspicion of PTB in 2017. The cutoff of 12 years old was selected based on similar cutoffs in the prediction algorithms selected. We used electronic medical records to search for patients where a sputum AFB smear and culture were ordered. If the sputum AFB was ordered with explicit clinical suspicion for evaluating non-TB mycobacteria (NTBM), then these patients were not included in our study. Patients were also excluded from analysis if they did not have a chest X-ray, as this was a requirement for all of the predictive algorithms. Patients who were treated empirically as culture-negative PTB were also excluded as we believed categorizing these patients as a confirmed disease would bias our study. We also excluded patients with a known diagnosis of active PTB before admission. Finally, patients with solely extra PTB, without considerations or investigations for PTB, were excluded from the analysis.

For the final set of patients, we recorded demographic, clinical, and radiographic information to assess the accuracy of the prediction algorithms outlined in the selected papers. Statistical analysis was performed using the Mann-Whitney test (also known as the Wilcoxon rank-sum test) for continuous nonpaired nonparametric variables (age, length of stay, and the number of sputum samples) and chi-square tests with Yates's correction for categorical variables.

We scored our patients using the criteria outlined in these various models to assess their accuracy in our population. Sensitivities, specificities, positive predictive values, and negative predictive values were obtained for each model using this data set. We then assessed the potential number of patient days of respiratory isolation saved for each model by summing the total number of days of respiratory isolation for each patient who would have been correctly de-escalated using the model. Similarly, we calculated the potential number of sputum samples saved.

## Results

We identified 244 patients placed in respiratory isolation for suspicion of PTB during the specified period. Ultimately, 36 patients were found to have PTB with an AFB smear-positive rate of 72.2% and 208 were found to be negative for PTB. Ten patients were found to have extra PTB, seven with coexisting PTB and three without PTB. One patient in the PTB-negative group with extra PTB had pleural TB.

The patients with PTB were older and more likely to be immigrants. Most immigrants in our population were of Caribbean ancestry. There was no statistical difference in the gender split between the two groups nor in the HIV positivity rate. The patients in the PTB group were more likely to have a cough, weight loss, and night sweats than the non-PTB patients, while hemoptysis and subjective fevers were not statistically significant (Table 1). The chest X-ray findings were highly clinically significant in distinguishing PTB from non-PTB; both upper lobe infiltrates and cavitary upper lobe disease were noted to have *P*-values <0.001.

**Table 1:** Patients' characteristics. ^a^*P*-values for categorical variables by chi-square tests with Yates's correction and continuous variables with the Mann-Whitney test. PTB, pulmonary tuberculosis; HIV, human immunodeficiency virus

	PTB negative (*N* = 208)	PTB positive (*N* = 36)	*P*-value^a^
Demographic			
Mean age (in years)	54.61	45.93	0.009
Male (%)	100 (48.8)	22 (61.1)	0.188
Immigrant (%)	43 (20.8)	28 (77.8)	<0.001
Caribbean	29	17	
Latin American	6	6	
Asian	2	2	
African	5	3	
Middle Eastern	3	0	
HIV positive (%)	38 (18.3)	7 (19.4)	0.968
Symptoms			
Cough (%)	104 (50)	25 (69.4)	0.042
Hemoptysis (%)	26 (12.7)	8 (22.2)	0.187
Subjective fever (%)	74 (36.1)	19 (52.8)	0.069
Weight loss (%)	49 (23.9)	27 (75)	<0.001
Night sweats (%)	26 (12.7)	15 (41.7)	<0.001
Chest X-ray			
Upper lobe infiltrate (%)	40 (19.2)	25 (69.4)	<0.001
Upper lobe cavitary disease (%)	12 (5.8)	12 (33.3)	<0.001

The patients with PTB had vastly different hospital courses and isolation experiences than the non-PTB patients. The median length of stay differed from 24 to 6 days, respectively. The patients with PTB were more likely to have been placed in respiratory isolation on admission (hospital Day 0), while many patients in the non-PTB group were placed in respiratory isolation after admission with a median time to respiratory isolation one day post admission (Table 2).

**Table 2:** Hospital outcomes. ^a^*P*-values for categorical variables by chi-square tests with Yates's correction and continuous variables with the Mann-Whitney test. ^b^*P*-values cannot be calculated due to errors associated with dividing by 0. ^c^This patient had Mycobacterium avium complex, a species of nontuberculosis mycobacterium ^d^Two patients had sputum culture positive for PTB, one patient with extra PTB in a lymph node. ^e^Extra PTB in PTB-negative group: two lymph nodes and one pleural TB. AFB, acid-fast bacilli; IQR, interquartile range; MTB, Mycobacterium tuberculosis; PTB, pulmonary tuberculosis; N/A, not applicable

	PTB negative (*N* = 208)	PTB positive (*N* = 36)	*P*-value^a^
Mean and median length of stay (days)	11.9 and 6.0	27.3 and 24.0	<0.001
Mean and median time from admission to isolation (IQR; days)	3.3 and 1 (0,3)	0.19 and 0 (0,0)	<0.001
Isolation on admission (%)	59 (28.8)	30 (76.9)	<0.001
Mean days in isolation	2.8	22.3	<0.001
Zero days in isolation (%)	61 (29.8)	0 (0%)	N/A^b^
Sputum sample per patient	1.3	9.5	<0.001
Zero sputum samples collected (%)	113 (54.3)	1 (2.8)	<0.001
Mean number of morning sputum samples	0.3	4.2	<0.001
Sputum AFB smear positive (%)	1 (0.5)	26 (72.2)	<0.001
First sputum AFB smear positive (%)	0 (0)	18 (50.0)	N/A^b^
First morning sputum AFB smear positive (%)	1 (0.5)^c^	19 (52.8)	<0.001
Morning sputum MTB culture positive (%)	0 (0)	25 (69.4)	N/A^b^
Bronchoscopy performed	10	4^d^	0.258
Extra PTB	3^e^	7	<0.001

The patients in the PTB-positive group were mostly AFB smear positive, with 72.2% having at least one smear-positive sputum and 50% were AFB smear positive on their first sputum sample. The morning sputum smear positivity in the PTB group was 52.8%, and 25 of the 26 (96%) patients with AFB smear-positive disease tested positive on at least one morning sputum sample. The morning sputum also proved to be diagnostic in 69.4% of the patients in the PTB group. There was one patient in the PTB-negative group who had an AFB-positive smear that was ultimately identified as NTBM (Table 2).

Bronchoscopy was performed 14 times, 10 in the PTB-negative group and 4 in the PTB-positive group. Of the four patients in the PTB group who underwent bronchoscopy, two ultimately had their sputum samples grow TB and one was found to have extra PTB with a culture-positive lymph node. None of the patients in the PTB-negative group who underwent bronchoscopy had extra PTB.

Out of the six studies that we analyzed, four had sensitivities above 90% and two [20,23] had sensitivities of 100% for our study population. The corresponding specificities were 50% and 20%, respectively. If these studies were utilized during the study period, they would have saved 275 and 157 patient days of respiratory isolation, respectively. They would have also theoretically saved 70 and 31 sputum samples, respectively (Table 3).

**Table 3:** Application of predictive models. PPV, positive predictive value; NPV, negative predictive value

	Sensitivity	Specificity	PPV	NPV	Number of isolation days saved	Number of sputum samples saved
Bock et al. [18]	0.92	0.69	0.34	0.98	344	113
El-Solh et al. [19]	0.95	0.29	0.19	0.97	185	37
Gaeta et al. [20]	1	0.50	0.26	1	275	70
Redd and Susser [21]	0.38	0.90	0.41	0.89	511	231
Tattevin et al. [22]	0.81	0.73	0.34	0.96	392	134
Wisnivesky et al. [23]	1	0.20	0.18	1	157	31
Rakoczy et al. [24]	0.89	0.50	0.24	0.96	251	85

## Discussion

The utility and impact of clinical-radiographic prediction models are highly variable. In our study, the most highly accurate model would have potentially saved 344 patient days of respiratory isolation and 113 sputum samples (Table 3). However, when building these models and defining their cutoff points, accuracy may not be the highest priority due to the severe consequences of missing cases of PTB. The number of sputum samples saved is not as large as the number of patient days saved from respiratory isolation for several reasons. Though the quickest recommended rate of sputum sample collection is every eight hours, in clinical practice, this is hardly common, especially in underfunded institutions, as sputum collection is limited by the ability of staff to collect samples and the patients’ capability and willingness to produce adequate sputum. Often, a patient will not be able to produce an adequate sputum sample more than once per day or may need assistance by way of induction with saline nebulizers. Therefore, a patient may stay in isolation without producing adequate sputum for sampling. Some patients are de-escalated from isolation before any sputum samples are collected due to a change in clinical status and suspicion of an alternate diagnosis.

We believe the gender difference, which is not statistically significant, maybe arbitrary due to the small sample size. The PTB group had 61% men, which is higher than reported by many other studies. Hemoptysis is classically associated with TB. In a group of general hospitalized patients, the incidence of hemoptysis is very low. Many physicians are aware of the association between hemoptysis and PTB, which likely leads to triggering clinical suspicion of PTB. We suspect this is the reason for the lack of statistical significance of the presence of hemoptysis between the PTB and non-PTB groups. This is also a likely explanation for the high occurrence of fever and cough in the non-PTB groups. If this set was compared with a generalized group of hospitalized patients, we would expect all these symptoms would be statistically significant.

Radiographic findings, as demonstrated in our study, continue to be an important and objective sign in identifying PTB. CT scans offer more information than chest X-rays, but it is debatable how much value they add to identifying TB. As CT scans are costlier and require transporting patients, we do not believe they will be additive; however, we did not attempt to answer this question in our study.

The great difference in length of hospitalization between patients with PTB and those without PTB arose mostly due to monitoring for seroconversion post initiation of anti-TB therapy (ATT), the threshold to discontinue respiratory isolation (and discharge the patient). This is also one reason for the high number of sputum samples collected per patient in the PTB-positive group. In addition, to discharge a patient with PTB, several public health measures needed to be instituted, including setting up direct observation therapy (DOT) of ATT. The second explanation for the high number of sputum samples in the PTB group is that physicians treating patients with AFB smear-negative disease may have high clinical suspicion for PTB and would often request additional sputum samples. AFB smear-negative disease remains an underappreciated clinical entity among many physicians with low clinical exposure to TB. As evident in our study, the number of cases of TB even in an urban safety net hospital in the United States servicing a large immigrant population is relatively low. As such, many internal medicine physicians training in the United States will develop a paucity of TB experience during their residency (typically three years).

Many of the patients in the PTB-negative group spent zero days in isolation (29.8%) and had zero sputum samples collected (54.3%). This may reflect changes in clinical suspicion based on a change in the treating physician. The emergency room physicians may often suspect a patient has PTB and admit them to an isolation bed, but then the care is transferred to a hospitalist, usually trained in internal medicine, family medicine, or pediatrics. If the attending physician has another explanation for the patient’s clinical presentation and a strong suspicion that PTB is unlikely, the isolation order will be discontinued and no further sputum samples will be collected. Conversely, many patients in the PTB-negative group were placed in respiratory isolation several days after admission (upper quartile three days after hospital admission). These patients are likely a group of patients for who the treating physician initially did not have high clinical suspicion for TB but later had no obvious diagnosis and PTB began to be included in the differential diagnosis. These observations may be a manifestation of differences in clinical training and priorities on behalf of the attending physicians in the emergency department and inpatient services.

Studies on the utility of bronchoscopy in identifying patients with PTB have been mixed. In one study, patients with high clinical suspicion of PTB who were unable to generate adequate sputum or have smear-negative sputum may have a high probability of smear positivity on bronchoalveolar lavage (BAL) [25]. Another study found the added value of bronchoscopy over induced sputum to be 15% in identifying smear-positive TB in patients with previous smear-negative sputum [26]. In our study, it influenced management in only one patient. Morning sputum samples and induced sputum samples may be of high yield in the diagnosis of PTB as they include respiratory secretions from the entire bronchial tree rather than the one segment, which would be sampled from a BAL. A meta-analysis of five studies comparing induced sputum to bronchoscopy (BAL and bronchial washing) found equivalent results in diagnosis for both culture and smear positivity [27]. In patients in which there is a high index of suspicion for PTB, the best course of action may be to begin treatment after obtaining three sputum samples and patiently await the final culture results. For these patients, polymerase chain reaction (PCR) testing may often be more aggressively pursued.

This study had several areas that may limit the interpretations. The retrospective nature of our study meant that we had to depend on clinical data present in the medical record to judge how patients should move through the decision trees. By having longer lengths of stays and being more likely to have been seen by pulmonary and infectious disease specialists, these patients had more clinical data available to review. Patients who were ultimately treated for culture-negative PTB represent a small subset of patients who were excluded (of which none were identified during our analysis). In future studies, it would be reasonable to include some of these patients in the TB-positive group on a case-by-case basis; however, we felt the ambiguity in their classification, which ultimately was at the discretion of the treating physician, would bias our analysis. The exclusion of patients without chest X-rays likely did not affect the data set as chest X-rays are easily and frequently acquired on almost all inpatients in our hospital, resulting in few, if any, patients being excluded.

In the future, we expect prediction models to have continued utility in hospitals with a low incidence of TB as these facilities are least likely to have on-site mycobacterial laboratory and PCR testing, limiting the timely diagnosis of PTB. These institutes are most likely to have the highest percentage of unnecessary isolation. Thus, most likely to benefit from prediction models. The number of prediction models available for infection control committees to choose from will likely create heterogeneity among individual hospitals’ guidelines. The Xpert MTB-RIF assay (Xpert Ultra, Cepheid, Sunnyvale, CA, USA) can both identify cases of TB and rifampin resistance in several hours and may decrease reliance on sputum AFB staining and cultures [28,29]. However, it is still lacking widespread use due to cost, which is a hurdle for developing countries that may not be able to afford it, and in developed countries with a low incidence of TB. Additional technologies being developed (urinary and serum biomarkers ) may offer rapid ruling out of TB testing at a lower cost than PCR, which may decrease the need for clinical-radiographic prediction models [1].

The outbreak of respiratory diseases, including H1N1 influenza and now the COVID-19 pandemic, compete for the resources needed to control TB. Balancing the prevention of nosocomial TB transmission and efficient allocation of resources requires careful consideration in the future and is an ongoing matter of public health.

## Conclusions

Clinical-radiographic predictive algorithms may offer an expedited way for clinicians to de-escalate respiratory isolation in low-risk patients. These algorithms are complimentary and not a substitution for clinical judgment. In patients with otherwise clinically suspicious TB and AFB smear negative, a clinical-radiographic predictive algorithm should not be used to withhold antimycobacterial treatments, which are potentially lifesaving. Due to the cumbersome nature of respiratory isolation, prediction models may still have a role to play in the equitable and efficient use of healthcare resources despite advances in molecular diagnostic technologies. Bronchoscopy has a limited role in the diagnosis of PTB and may be obviated in the future by emerging diagnostic technologies.

## References

[1] Dheda K, Barry CE, 3rd 3rd, Maartens G (2016). Tuberculosis. Lancet.

[2] Jensen PA, Lambert LA, Iademarco MF, Ridzon R (2005). Guidelines for preventing the transmission of Mycobacterium tuberculosis in health-care settings, 2005. MMWR Recomm Rep.

[3] Blumberg HM, Watkins DL, Berschling JD (1995). Preventing the nosocomial transmission of tuberculosis. Ann Intern Med.

[4] Tran K, Bell C, Stall N (2017). The effect of hospital isolation precautions on patient outcomes and cost of care: a multi-site, retrospective, propensity score-matched cohort study. J Gen Intern Med.

[5] Guilley-Lerondeau B, Bourigault C, Guille des Buttes AC, Birgand G, Lepelletier D (2017). Adverse effects of isolation: a prospective matched cohort study including 90 direct interviews of hospitalized patients in a French University Hospital. Eur J Clin Microbiol Infect Dis.

[6] Wisnivesky JP, Serebrisky D, Moore C, Sacks HS, Iannuzzi MC, McGinn T (2005). Validity of clinical prediction rules for isolating inpatients with suspected tuberculosis. A systematic review. J Gen Intern Med.

[7] Solari L, Acuna-Villaorduna C, Soto A, van der Stuyft P (2011). Evaluation of clinical prediction rules for respiratory isolation of inpatients with suspected pulmonary tuberculosis. Clin Infect Dis.

[8] Kanaya AM, Glidden DV, Chambers HF (2001). Identifying pulmonary tuberculosis in patients with negative sputum smear results. Chest.

[9] Mello FC, Bastos LG, Soares SL (2006). Predicting smear negative pulmonary tuberculosis with classification trees and logistic regression: a cross-sectional study. BMC Public Health.

[10] Soto A, Solari L, Agapito J, Acuna-Villaorduna C, Lambert ML, Gotuzzo E, van der Stuyft P (2008). Development of a clinical scoring system for the diagnosis of smear-negative pulmonary tuberculosis. Braz J Infect Dis.

[11] Wang CS, Chen HC, Chong IW (2008). Predictors for identifying the most infectious pulmonary tuberculosis patient. J Formos Med Assoc.

[12] Soto A, Solari L, Díaz J, Mantilla A, Matthys F, van der Stuyft P (2011). Validation of a clinical-radiographic score to assess the probability of pulmonary tuberculosis in suspect patients with negative sputum smears. PLoS One.

[13] Pinto LM, Dheda K, Theron G (2013). Development of a simple reliable radiographic scoring system to aid the diagnosis of pulmonary tuberculosis. PLoS One.

[14] Aguilar J, Yang JJ, Brar I (2009). Clinical prediction rule for respiratory isolation of patients with suspected pulmonary tuberculosis. Infect Dis Clin Pract.

[15] Scott B, Schmid M, Nettleman MD (1994). Early identification and isolation of inpatients at high risk for tuberculosis. Arch Intern Med.

[16] Yeh JJ, Neoh CA, Chen CR, Chou CY, Wu MT (2014). A high resolution computer tomography scoring system to predict culture-positive pulmonary tuberculosis in the emergency department. PLoS One.

[17] Tessema TA, Bjune G, Assefa G, Bjorvat B (2001). An evaluation of the diagnostic value of clinical and radiological manifestations in patients attending the addis ababa tuberculosis centre. Scand J Infect Dis.

[18] Bock NN, McGowan JE, Jr Jr, Ahn J, Tapia J, Blumberg HM (1996). Clinical predictors of tuberculosis as a guide for a respiratory isolation policy. Am J Respir Crit Care Med.

[19] El-Solh A, Mylotte J, Sherif S, Serghani J, Grant BJ (1997). Validity of a decision tree for predicting active pulmonary tuberculosis. Am J Respir Crit Care Med.

[20] Gaeta TJ, Webheh W, Yazji M, Ahmed J, Yap W (1997). Respiratory isolation of patients with suspected pulmonary tuberculosis in an inner-city hospital. Acad Emerg Med.

[21] Redd JT, Susser E (1997). Controlling tuberculosis in an urban emergency department: a rapid decision instrument for patient isolation. Am J Public Health.

[22] Tattevin P, Casalino E, Fleury L, Egmann G, Ruel M, Bouvet E (1999). The validity of medical history, classic symptoms, and chest radiographs in predicting pulmonary tuberculosis: derivation of a pulmonary tuberculosis prediction model. Chest.

[23] Wisnivesky JP, Kaplan J, Henschke C, McGinn TG, Crystal RG (2000). Evaluation of clinical parameters to predict Mycobacterium tuberculosis in inpatients. Arch Intern Med.

[24] Rakoczy KS, Cohen SH, Nguyen HH (2008). Derivation and validation of a clinical prediction score for isolation of inpatients with suspected pulmonary tuberculosis. Infect Control Hosp Epidemiol.

[25] Sanjeevaiah S, Haranal MY, Buggi S (2018). Role of flexible bronchoscopy in patients with sputum-negative pulmonary tuberculosis. Indian J Thorac Cardiovasc Surg.

[26] Rao GN, Venu M, Rani NU, Sravani M (2016). Induced sputum versus bronchial washings in the diagnosis of sputum negative pulmonary tuberculosis. J Family Med Prim Care.

[27] Luo W, Lin Y, Li Z, Wang W, Shi Y (2020). Comparison of sputum induction and bronchoscopy in diagnosis of sputum smear-negative pulmonary tuberculosis: a systemic review and meta-analysis. BMC Pulm Med.

[28] Dorman SE, Schumacher SG, Alland D (2018). Xpert MTB/RIF Ultra for detection of Mycobacterium tuberculosis and rifampicin resistance: a prospective multicentre diagnostic accuracy study. Lancet Infect Dis.

[29] Menon LJ, Feliciano CS, de Campos MR, Bollela VR (2020). Decision making to discharge patients from airborne infection isolation rooms: the role of a single GeneXpert MTB/RIF strategy in Brazil. Infect Control Hosp Epidemiol.

